# Heme Oxygenase-1 Expression as a Prognostic Marker in Early-Stage HCC Undergoing Resection or Liver Transplantation

**DOI:** 10.3390/life15101589

**Published:** 2025-10-11

**Authors:** Ramona Cadar, Alin Mihai Vasilescu, Ana Maria Trofin, Alexandru Grigorie Nastase, Mihai Zabara, Cristina Muzica, Corina Lupascu Ursulescu, Mihai Danciu, Andrei Pascu, Iulian Buzincu, Delia Ciobanu, Ianole Victor, Cristian Dumitru Lupascu

**Affiliations:** 1 Surgical Department, Grigore T. Popa University of Medicine and Pharmacy, 700115 Iasi, Romania; tibaramona@yahoo.com (R.C.); alex.gr.nastase@gmail.com (A.G.N.); lungu.christina@yahoo.com (C.M.); corina.ursulescu@gmail.com (C.L.U.); mihai.danciu@umfiasi.ro (M.D.); delia.ciobanu@umfiasi.ro (D.C.);; 2 General Surgery and Liver Transplant Clinic, St. Spiridon University Hospital, 700111 Iasi, Romania; 3 Institute of Gastroenterology and Hepatology, Sf. Spiridon University Hospital, 700111 Iasi, Romania; 4 Radiology Clinic, St. Spiridon University Hospital, 700111 Iasi, Romania; 5 Departament of Pathology, St. Spiridon University Hospital, 700111 Iasi, Romania; 6 Anesthesiology and Intensive Care Department, Clinical Emergency County Hospital “St. Spiridon” Iasi, 700111 Iasi, Romania; 7 Departament of Pathology, Prof. dr. N. Oblu University Hospital, 700111 Iasi, Romania

**Keywords:** hepatocellular carcinoma, HO-1, biomarker, prognostic

## Abstract

**Background:** Hepatocellular carcinoma (HCC) is a prevalent malignancy with high mortality, often arising in the context of chronic liver diseases. Heme oxygenase-1 (HO-1), an inducible enzyme involved in heme degradation, has been implicated in both hepatoprotection and tumor progression. This study evaluates the expression of HO-1 in HCC and its association with clinicopathological features and patient survival. **Materials and Methods:** We retrospectively analyzed 58 HCC cases diagnosed between 2018 and 2023 at “Sf. Spiridon” Emergency County Hospital, Iasi. HO-1 expression was assessed immunohistochemically and quantified using a semi-quantitative immunoreactivity score (IRS). Statistical correlations between HO-1 expression and clinical, pathological, and survival parameters were evaluated using univariate analysis, ROC curves, and Kaplan–Meier survival models. **Results:** High HO-1 expression (IRS > 1) was significantly associated with hepatitis C virus etiology (*p* = 0.004, V = 0.381), vascular invasion (*p* = 0.019, V = 0.309) and perioperative anticoagulant therapy (*p* = 0.007, V = 0.352). However, HO-1 expression did not correlate with overall survival (OS). In contrast, solid growth pattern (*p* = 0.030) and serum α-fetoprotein levels of 10–99 ng/mL (*p* = 0.022) were negatively associated with OS. **Conclusions:** HO-1 expression in HCC was found to be associated with vascular invasion, but not with overall survival. While this may indicate a potential link to certain aggressive tumor features, the overall role of HO-1 in HCC biology remains unclear. These findings suggest that HO-1 should be considered an exploratory rather than definitive prognostic marker, and further research is warranted to clarify its function and potential utility, including investigation of its detectability in biological fluids for non-invasive monitoring.

## 1. Introduction

Hepatocellular carcinoma (HCC) is an epithelial neoplasia and is a major health problem, ranking 3rd as a cause of cancer mortality and 6th in global incidence [1]. About 90% of liver tumors are hepatocellular carcinoma. Histologically, it encompasses forms including trabecular/sinusoidal, pseudoglandular, solid and undifferentiated [2,3].

Global prevalence varies depending on the etiology and the prevalence of liver diseases in different regions of the world. In some parts of the world, a reduction in the incidence of HCC is expected with improvements in hepatitis B virus (HBV) and hepatitis C virus (HCV) therapy [4]. However, the risk of HCC is not eliminated in patients treated for HBV-HCV, and with the increasing burden of Nonalcoholic fatty liver disease (NAFLD), the global incidence of HCC is expected to rise in the coming years. Additionally, HCC screening programs do not ensure adequate diagnostic accuracy [5].

Nonetheless, it has been demonstrated that HCC may arise without cirrhosis, particularly in the setting of chronic HBV and HCV infections, or in conjunction with variables linked to metabolic syndrome, such as nonalcoholic fatty liver disease, nonalcoholic steatohepatitis, obesity, and insulin resistance. The likelihood of obtaining HCC is 15–20 times greater in those infected with HBV compared to the general uninfected population [6].

A number of studies have pointed to the importance of cancer cell viability, which is probably linked to the increased presence of protective proteins. These include inhibitors of apoptosis proteins (IAPs) and heme oxygenase-1 (HO-1) [7].

Heme oxygenases (HOs) are stress-responsive enzymes that catalyze heme degradation into biliverdin/bilirubin, carbon monoxide (CO), and ferrous iron [8,9]. In mammals, two isoforms exist: HO-1 (inducible) and HO-2 (constitutive); a putative HO-3 is not established in humans [10,11]. HO-1 is transcriptionally induced by oxidative, inflammatory, hypoxic, and oncogenic signals (e.g., Nrf2, STAT3) and modulates redox homeostasis, inflammation, angiogenesis, and cell survival [12].

HO-1 exhibits a context-dependent, dual role. Physiologically, its induction confers cytoprotection against oxidative injury, dampens inflammation, and supports tissue remodeling [13]. Conversely, in malignant settings, aberrant or sustained HO-1 activation can promote tumor progression by enhancing invasive behavior, facilitating immune evasion, and fostering therapy tolerance through oxidative-stress buffering and metabolic reprogramming [14].

Modulation of HO-1 has broad hepatic and oncologic effects. In the liver, inducing or overexpressing HO-1 influences acute and chronic inflammation, accelerates or mitigates fibrogenesis, alters susceptibility to apoptotic injury, and affects HBV/HCV replication; it also protects liver grafts against ischemia–reperfusion damage after transplantation or hemorrhage/resuscitation [15,16].

The endogenous regulation of HO-1 expression during HCV infection has been a subject of debate. While some studies reported increased HO-1 and decreased Bach-1 expression in HCV-replicating hepatoma cells [17], other research found reduced HO-1 expression in cell lines expressing the HCV core protein [18]. This latter observation is supported by evidence indicating that the HCV core protein interferes with HO-1 induction by heme, heavy metals, and peroxides [19].

Further investigations into microRNAs have clarified HO-1’s impact on HCV replication. miR-122 seems crucial for HCV RNA replication [20]. This is evidenced by an antagomir targeting miR-122 reducing HCV RNA levels in replicon cell lines while transfecting a 2’-O-methyl-mimic miR-122 increased HCV levels up to 2.5-fold [21]. The antagomir also lowered Bach-1 and elevated HO-1 mRNA levels, suggesting a mechanism for miR-122’s effects on HCV replication. This finding is reinforced by a report demonstrating that miR-196 could downregulate the HO-1 transcriptional repressor Bach-1, thereby upregulating HO-1 expression [22].

Because HO-1 sits at the intersection of stress and oncogenic signaling, we evaluated its expression in early-stage HCC treated with curative intent, testing associations with clinicopathology and survival.

## 2. Materials and Methods

### 2.1. Study Design and Patient Allocation

This retrospective study initially considered 75 patients diagnosed with hepatocellular carcinoma (HCC) who underwent liver resection or liver transplantation between January 2018 and December 2023 at “Sf. Spiridon” Emergency County Hospital, Iași, Romania. Eligible patients were aged 18 years or older, classified as Barcelona Clinic Liver Cancer (BCLC) stage 0, A, or B, and included those who received bridging therapy with transarterial chemoembolization (TACE) prior to surgery. Only cases with a confirmed histopathological diagnosis of HCC were included.

Patients were excluded if they were younger than 18 years, had advanced disease (BCLC stage C or higher), or if histopathological slides could not be retrieved for logistical reasons.

The study was approved by the Ethics Committee of “Sf. Spiridon” Emergency County Hospital (No. 479/7 October 2024), and written informed consent was obtained from all patients.

### 2.2. Study Groups

After applying the exclusion criteria, the final study group consisted of 58 patients diagnosed with hepatocellular carcinoma between 2018 and 2023 at “Sf. Spiridon” Emergency County Hospital, Iași, Romania.

The control group included 20 samples of normal liver tissue obtained from patients who underwent liver biopsy for presumed but not confirmed hepatopathy.

### 2.3. Immunohistochemistry

All tumor samples were routinely processed by fixation in 10% neutral buffered formalin and paraffin embedding. The control group included 20 samples of normal liver tissue obtained by biopsy from patients with non-tumor pathology. Immunohistochemical tests were performed on 4 µm thick sections using an anti-HO-1 monoclonal antibody (clone HO-1-1, mouse anti-human, 1:200, ab13248, Abcam, Cambridge, UK) after pretreatment (HIER—heat-induced epitope retrieval) with a specific epitope retrieval solution (pH 9).

The detection of the immunoreaction was performed using the Mouse and Rabbit Specific HRP/DAB Detection IHC Kit (ab64264, Abcam, Cambridge, UK). To check the specificity of the immunoreactivity, the primary antibody was omitted and replaced with non-immunized serum at the same dilution (negative control). The immunohistochemistry tests were performed on two replicates from each sample [23].

### 2.4. Immunohistochemical Assessment Method

**Figure 1:**
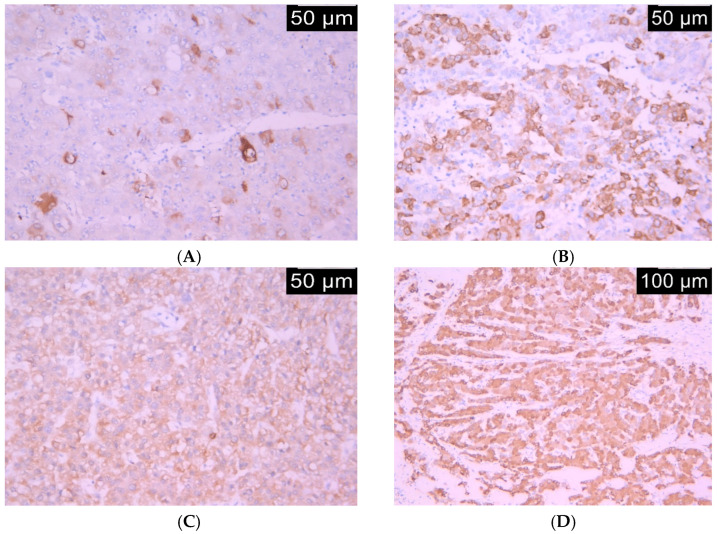
The hepatocellular carcinoma HO-1 unicellular coloration (**A**), IHC 20×, HO-1 mosaic coloration (**B**), IHC 20×. Were diffusely positive for HO-1 low intensity (**C**), IHC 20× and HO-1 moderate intensity (**D**), IHC 10×.

Two independent investigators blindly assessed HO-1 expression. Expression of HO-1 was defined as the presence of cytoplasmic staining in tumor cells. A semi-quantitative four-tiered scoring system was used to measure the proportion of stained tumor cells, as follows: 0 = no positive tumor cells; 1 = <30% of tumor cells; 2 = 30–60%; and 3 = >60%. The intensity of immunoreaction was evaluated using a four-tiered system: 0—negative, 1—weak, 2—moderate, 3—strong (Figure 1). Discordant results were re-evaluated “in panel” to achieve consensus. An immunoreactivity score (IRS) was calculated by multiplying the proportion and the intensity scores, respectively. As a result, IRS ranged from 0 to 6.

### 2.5. Statistical Analysis

The statistical analysis was conducted with the help of IBM Statistical Package for the Social Sciences (SPSS) version 26 and MedCalc version 23.2.0.

The chi-squared test and Fisher exact test were computed for univariate analysis of HO-1 expression and clinicopathological parameters (gender, age, hepatitis C virus, hepatitis B virus, toxic etiology, serum α-fetoprotein, tumor size, tumor number, Barcelona Clinic Liver Cancer grading, perioperative anticoagulant therapy, antiviral therapy, perioperative transcatheter arterial chemoembolization, postoperative transcatheter arterial chemoembolization, chemotherapy, trabecular growth pattern, solid growth pattern, pseudoglandular growth pattern, histological grade, tumor stage, lymphatic invasion, vascular invasion, and liver cirrhosis).

Furthermore, Cramer’s V was used to examine the association between two categorical variables (negligible: 0.00–0.10; weak: 0.10–0.20; moderate: 0.20–0.40; relatively strong: 0.40–0.60; strong: 0.60–0.80; very strong: 0.80–1.00) [24].

Overall survival (OS) was considered as the period of time that passed from the pathological diagnosis to the patient’s death. For the estimation of OS, the Kaplan–Meier curve (log-rank test) was used. To calculate the hazard ratios and confidence intervals for OS, the Cox proportional hazard model was used in an univariable and multivariable manner. A *p*-value < 0.05 was considered statistically significant. Please see Appendix A.

## 3. Results

### 3.1. Clinicopathological Parameters and HO-1 Expression in the Study Group

Fifty-eight patients with HCC were included in the analysis. The cohort comprised 49 men (84.5%) and 9 women (15.5%), with a mean age of 63.8 +/− 11.13 years (range 30–81 years). Five patients (8.6%) were younger than 50 years, and 53 patients (91.4%) were 50 years or older. The main etiologies of underlying liver disease were hepatitis C virus (HCV) infection in 33 cases (56.9%), hepatitis B virus (HBV) infection in 6 cases (10.3%), and toxic etiology in 7 cases (12.1%); some patients had multiple etiological factors. Liver cirrhosis was present in 40 patients (69.0%) (Table 1).

The correlations between HO-1 expression and clinicopathological parameters are shown in Table 2.

HO-1 immunohistochemistry revealed low expression in 42 cases (72.4%) and high expression in 16 cases (27.6%).

HO-1 expression did not significantly differ by gender (*p* = 0.696) or age group (*p* = 0.609). 

A significant statistical correlation was identified between HO-1 expression and VHC etiology (*p* = 0.004), suggesting that a high expression of HO-1 is significantly more associated with HCV-positive cases. Additionally, an effect size was calculated (V = 0.381) that is considered to have moderate practical significance. No significant correlations were found with HBV or alcohol-related etiologies.

Neither tumor size nor tumor number alone showed statistical significance in relation to the studied outcome (*p* > 0.05). Similarly, BCLC stage, serum AFP levels, antiviral therapy, perioperative TACE, postoperative TACE, and chemotherapy did not show significant differences in HO-1 status.

However, the Milan criteria, which incorporate both parameters, were associated with improved outcomes (*p* = 0.03). Low expression was more associated with Milan 0 with a moderate effect size (0.275).

Regarding the association between HO-1 expression and preoperative anticoagulant therapy, a statistically significant correlation was noted (*p* = 0.007), with an effect size of V = 0.352. This result highlights that a high expression of HO-1 is associated with the administration of anticoagulant therapy preoperative, although its practical significance is moderate.

The correlations between HO-1 expression and histological parameters are provided in Table 3.

A statistically correlation was found between HO-1 expression and vascular invasion (*p* = 0.02) but showed no association with lymphatic invasion or cirrhosis status. In this study was observed that high expression of HO-1 is more associated with the presence of vascular invasion. The computed effect size (V = 0.309) showed moderate practical significance.

The pseudoglandular growth pattern was observed in 41 cases, being more frequent in the unfavorable outcome group (33 cases, 56.9%) compared to the favorable outcome group (8 cases, 13.8%) (*p* = 0.05, Cramer’s V = 0.281), while trabecular and solid growth patterns were not significantly related. Histological grade and overall tumor stage also showed no significant associations.

In order to evaluate HO-1 expression as low/high, a cutoff point for the IRS was considered after using a receiver operating characteristic (ROC) curve and calculating the Youden (J) index (Table 4) [25].

**Figure 2:**
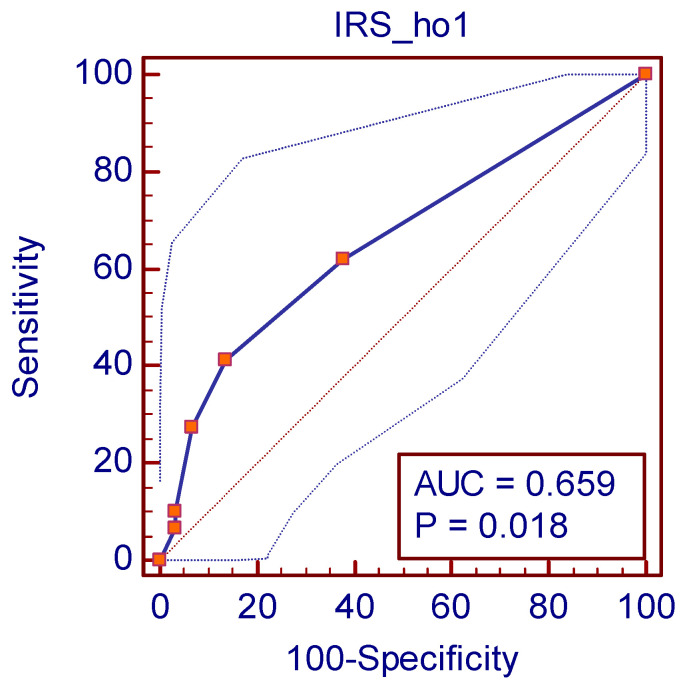
Aspect of statistically significant ROC curve.

By calculating the Youden (J) index (0.2759) (Table 1), a cutoff point was established as >1. Therefore, HO-1 high expression was defined as IRS > 1 and HO-1 low expression as IRS ≤ 1. The analysis revealed an AUC of 0.659 (*p* = 0.018), indicating a fair discriminative ability of HO-1 expression. Although the predictive power is not strong, the statistically significant *p*-value suggests that HO-1 expression contributes to outcome differentiation (Figure 2).

### 3.2. Survival and HO-1 Expression in the Study Group

Overall survival (OS) was defined as the time elapsed between histopathological diagnosis and death.

**Figure 3:**
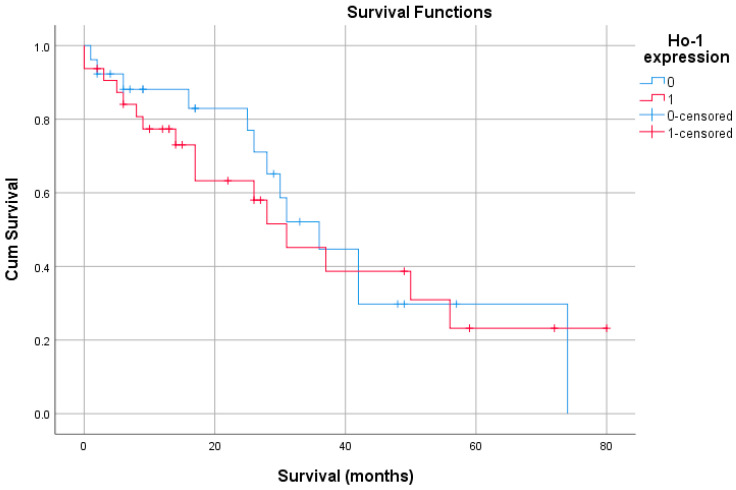
Kaplan–Meier overall survival (OS) by tumoral HO-1 expression. Cumulative survival curves over follow-up (months) are shown for patients with low HO-1 (IRS ≤ 1, blue) and high HO-1 (IRS > 1, red). Cross marks denote censored observations. Group differences were tested with the log-rank test; the legend indicates event and censoring for each group. OS = overall survival; HO-1 = heme oxygenase-1; IRS = immunoreactivity score.

Kaplan–Meier univariate survival analysis found no statistically significant association between HO-1 expression and OS (Figure 3). Furthermore, the univariable Cox proportional hazard found no significant correlation between HO-1 expression and OS. Therefore, a multivariable survival analysis using Cox proportional hazard could not be computed.

Patients in the negative HO-1 expression group had a slightly longer average survival compared to the positive group, but the difference (~3 months) is small. The overall survival for the entire cohort is around 38.6 months (Table 5).

In the overall cohort (N = 58), HO-1 expression showed no linear association with either endpoint: overall survival (r = −0.061, *p* = 0.650) or disease recurrence (r = 0.014, *p* = 0.915). Likewise, overall survival and disease recurrence were not linearly correlated (r = −0.047, *p* = 0.728). These near-zero coefficients indicate no detectable correlation between HO-1 and time-to-event outcomes in this dataset (Table 6).

## 4. Discussion

In this single-centre cohort of early-stage HCC treated with curative intent (resection or transplantation), HO-1 overexpression correlated strongly with vascular invasion and with HCV aetiology but did not independently predict overall survival. Tumor size or number alone were not prognostic, whereas the Milan criteria—which integrate both—retained expected prognostic value. A correlation between HO-1 expression and perioperative anticoagulant use likely reflects higher thrombotic risk in HO-1-high, invasion-prone tumors rather than a direct treatment effect.

Hepatocellular carcinoma is one of the most common types of cancer worldwide and shows a continuously increasing death rate, by approximately 2–3% per year, due to late diagnosis and the absence of curative treatment for advanced stages of the disease [26].

HO-1, the inducible heme-oxygenase isoform, is upregulated by diverse stressors—heavy metals, endotoxins, cytokines, heme, hypoxia, nitric oxide, and ultraviolet irradiation [27]—and contributes to cell proliferation, migration, and metastasis [28]. Nuclear localization of HO-1 has been associated with tumor progression and chemoresistance, although results are not uniform across studies; a truncated nuclear HO-1 variant was recently detailed [29]. Prognostically, increased HO-1 has been linked to improved survival in colorectal, gastric, small-intestinal adenocarcinoma, and oral squamous carcinoma, and subcellular distribution appears relevant based on data from head and neck, breast, and colorectal cancers [30,31]. Overall, HO-1 shows a dualistic role—protective in non-malignant contexts (limiting injury and fibrosis) yet tumor-promoting in established cancers, where antioxidant and anti-apoptotic effects can favour survival and progression [32].

In viral liver disease, HO-1 biology is etiologic-dependent. Studies on liver sections from HCV-infected patients indicate complex interactions between heme-enzymatic products and HCV; some report reduced HO-1 in hepatocytes harbouring HCV, while others show marked increases in autoimmune hepatitis and chronic HBV [18,33,34]. Our finding of elevated HO-1 in HCV-related HCC aligns with the chronic oxidative stress milieu in HCV and induction of cytoprotective enzymes as an adaptive response [35]. HO-1 upregulation in HCV-replicating hepatocytes may protect against viral-induced oxidative injury and can exert antiviral effects [19], yet HCV proteins can also interfere with HO-1 regulation, suggesting a selective pressure that favours HO-1–overexpressing tumor clones [36]. This may help explain the stronger association we observed in HCV versus HBV, consistent with reports that viral proteins (e.g., core, NS5A) activate Nrf2-dependent pathways to upregulate HO-1 [37,38], although the precise HCV-specific mechanism remains speculative [39]. Conversely, some HBV-related data suggest tumor-suppressive facets of HO-1; for example, PLC/PRF/5 cells (higher HO-1) show shorter doubling times than Hep3B, and increased HO-1 can reduce migration and invasion in Hep3B cells [8].

These mechanisms may partially explain why HO-1 overexpression was preferentially observed in HCV-related HCC in our cohort, despite the absence of a similar correlation with HBV. However, the precise biological basis for this HCV-specific association remains speculative [39]. We acknowledge this as a limitation of our study and propose that future research should investigate HO-1 expression at both transcriptional and post-translational levels in HCV- versus HBV-related HCC, along with functional analyses to clarify its role in viral oncogenesis and therapy response.

Individually, neither maximum tumor size nor tumor count significantly affected prognosis in our cohort. However, the Milan criteria, which integrate both parameters, were significantly associated with improved outcomes. This emphasizes that composite indices capturing total tumor burden provide more accurate prognostic stratification than single measurements. Although HO-1 expression did not correlate directly with these parameters, its association with vascular invasion suggests that HO-1 could serve as a complementary marker, identifying tumors with invasive potential even among those meeting Milan criteria.

Findings indicated that elevated cytosolic HO-1 levels were associated with lower tumor grade and better differentiation, showing no connection to invasiveness. Conversely, nuclear HO-1 localization was linked to higher tumor grade and poorer differentiation [40,41]. These findings are compelling and shed light on the conflicting roles of HO-1 in tumor progression, suggesting that its pro- or antitumor effects might hinge on its subcellular location and enzymatic capability [40,42]. The pseudo glandular growth pattern was observed in 41 tumors and was significantly more frequent in the unfavorable outcome group.

Subsequent research has revealed an association between HO-1 overexpression in replicon cells and increased resistance to oxidant-induced cytotoxicity, which may be responsible for resistance to chemotherapy treatment [35,36,43].

An additional observation in our cohort was the association between HO-1 and anticoagulant therapy. HO-1 byproducts can attenuate endothelial activation and platelet aggregation, suggesting a compensatory anti-thrombotic effect [44]. Prior work in aortic aneurysms noted linear regression of HO-1 expression with anticoagulation [44], yet clinical data do not support routine anticoagulation for HCC with macrovascular invasion due to bleeding risk and absent survival benefit [45]. These seemingly contradictory findings may imply a dual role for HO-1: a protective effect in non-tumoral tissues by reducing inflammation, and a pro-tumoral effect in HCC cells, where HO-1 upregulation may promote proliferation, angiogenesis, and metastasis [46,47].

Most importantly, our strong correlation between HO-1 and vascular invasion reinforces HO-1 as an active modulator of metastatic potential rather than a passive stress marker [45,48,49,50,51,52,53]. From a therapeutic perspective, targeting HO-1 or downstream effectors could help limit dissemination [54], and experimental HO-1 inhibition has sensitized tumors to chemotherapy and reduced metastasis [55].

Another novel finding of our study is the strong correlation between HO-1 overexpression and vascular invasion in HCC. Vascular invasion is a hallmark of tumor aggressiveness, predicting intrahepatic spread, early recurrence, and poor prognosis [48].

Although HO-1 overexpression was associated with aggressive features, it was not an independent predictor of survival. This suggests that its prognostic role may be context-dependent or mediated through other factors. Nevertheless, HO-1’s enzyme activity and its multifaceted involvement in tumor biology make it an attractive therapeutic target. Experimental inhibition of HO-1 has shown promise in sensitizing tumors to chemotherapy and reducing metastasis [55].

Mechanistically, HO-1 can promote invasion and metastasis through angiogenesis (co-promoter VEGF), migration, and possible EMT contributions [49,50], and facilitate immune evasion with increased tumor viability [45,51,52,53]. Functionally, chemoresistance has been linked to HO-1: replicon models with HO-1 overexpression resist oxidant-induced cytotoxicity [35,36], and across malignancies (renal, prostate, pancreatic cancers, melanoma, lymphosarcoma, hepatoma) HO-1 upregulation is tied to progression and treatment resistance [56,57,58,59]. Tumors with high HO-1 often show reduced sensitivity to agents such as etoposide, doxorubicin, cisplatin, and gemcitabine; conversely, HO-1 inhibition or silencing increases ROS, enhances cytotoxicity, suppresses growth in vivo, and can curb proliferation in gemcitabine-resistant pancreatic models [60,61]. In hematologic disease, elevated HO-1 in AML (HL-60R) correlates with reduced sensitivity to cytarabine/daunorubicin [60].

### 4.1. Clinical Implications

Emerging evidence links aberrant HO-1 activation to broad chemoresistance phenotypes via antioxidant buffering, metabolic reprogramming, and modulation of apoptosis and autophagy. Across multiple tumor types, HO-1 upregulation has been associated with reduced efficacy of cytotoxics and targeted agents, while HO-1 inhibition or knockdown can resensitize cells to therapy [62]. In HCC, repeated phase II/III disappointments of systemic agents that target single pathways underscore the complexity of resistance biology. For example, early signals with c-MET inhibition in selected patients did not translate into consistent survival benefit in later trials, despite compelling preclinical rationale [59].

Mechanistically, HO-1 can be induced downstream of oncogenic and stress-activated transcriptional programs (e.g., Nrf2, STAT3), and prior data suggest that HO-1 may be upregulated by oncogenes including c-MET, potentially enabling redox adaptation and survival in hypoxic, inflamed tumor microenvironments [38,62]. Our finding that HO-1 overexpression tracks with vascular invasion supports a model in which HO-1 contributes to an invasive, therapy-tolerant state.

Translational next steps include validating whether HO-1–high HCC exhibits reduced responsiveness to standard systemic regimens; testing rational combinations that co-target MET signalling and the HO-1/Nrf2 axis; and evaluating circulating HO-1 as a minimally invasive pharmacodynamic marker to monitor pathway suppression and resistance evolution. These hypotheses remain exploratory but are aligned with our observed pathology–HO-1 associations.

### 4.2. Strengths and Limitations of the Study

This study contributes regional data on HO-1 expression in surgically treated HCC and its association with clinicopathological parameters, including a novel link with vascular invasion. However, etiology is a major limitation: nearly all cases were HBV/HCV-related, with minimal representation of non-viral HCC (alcohol-related disease, NAFLD/NASH). Given that HCC etiology can shape oncogenic and stress-response pathways, including those that regulate HO-1, our findings should not be generalized to non-viral HCC without external validation. The modest sample size further limits power for survival modeling.

Nevertheless, several limitations should be acknowledged. The study cohort consisted of 58 patients, representing all eligible surgical HCC cases from our center over a five-year period. While this reflects a real-world population, the modest sample size limits statistical power—particularly for survival analyses—and may affect generalizability. The control group was derived from liver biopsy samples initially suspected of hepatopathy but ultimately reported as histologically normal. This pragmatic approach was ethically justified but carries the risk that subclinical inflammation influenced baseline HO-1 expression, potentially reducing observed differences.

Furthermore, HO-1 assessment relied on semi-quantitative IHC scoring, which—despite standardized procedures—remains partly subjective. The use of an ROC-derived cutoff (IRS > 1, AUC = 0.659) highlights the exploratory nature of our analysis, as this threshold only modestly distinguished between groups. Finally, due to the lack of statistical significance for HO-1 in univariable Cox regression, multivariable survival analysis could not be performed, preventing evaluation of HO-1 as an independent prognostic factor. Moreover, as an observational study, our findings reveal associations but cannot establish causality or the underlying biological mechanisms.

Our findings contribute to the limited data on HCC biomarkers from Eastern Europe, a region with distinct epidemiology characterized by a high prevalence of HCV. In this context, HO-1 overexpression appears to reflect an aggressive HCC phenotype shaped by chronic viral infection. This insight may help refine prognostic assessments and guide therapeutic decisions in Romanian patients. As HCV prevalence declines, it will be important to monitor how HO-1’s role evolves in relation to shifting etiologies. Larger multicenter studies are warranted to validate our findings and explore potential clinical applications of HO-1 as a biomarker or therapeutic target.

### 4.3. Future Perspectives

Despite these limitations, this study serves as an important starting point by characterizing HO-1 expression in a Romanian HCC cohort and highlighting its association with HCV infection and vascular invasion. These observations provide a basis for further research into HO-1 as a biological marker of tumor aggressiveness in specific etiological contexts. Future investigations should focus on validating these associations in larger, multicenter cohorts to strengthen statistical power and improve external validity. The selection of better-defined control groups, such as liver tissue from living donors or patients without chronic liver disease, would allow a clearer assessment of baseline HO-1 expression. Quantitative techniques, including digital pathology and molecular assays, should be employed to enhance the reproducibility of HO-1 measurement. In parallel, experimental studies using cellular and animal models are needed to unravel the mechanisms underlying HO-1 upregulation in HCV-related HCC and its potential contribution to vascular invasion. Moreover, exploring circulating HO-1 levels may offer a non-invasive approach to evaluate its clinical utility. Finally, HO-1 should be assessed as part of multi-marker panels rather than as a standalone factor, as its moderate association suggests it may complement established prognostic parameters. Overall, this work provides a valuable foundation for future studies aiming to clarify the biological and clinical significance of HO-1 in hepatocellular carcinoma.

## 5. Conclusions

HO-1 overexpression in HCC is intricately linked to HCV-associated tumor biology and features of aggressiveness such as vascular invasion and pseudo glandular growth patterns. Although it does not independently predict survival, HO-1 identifies a biologically invasive phenotype that may benefit from intensified surveillance and potentially targeted interventions. Its correlation with anticoagulant therapy underscores its relevance to tumor-associated coagulation pathways but also highlights the complexity of translating this into clinical benefit. These findings, grounded in a Romanian cohort, underscore the importance of integrating molecular and clinical factors for a comprehensive understanding of HCC prognosis and therapy. In light of emerging data connecting HO-1 to therapy resistance and oncogenic c-MET signaling, prospective studies should test whether HO-1 status refines patient selection for systemic therapies and whether dual targeting of MET and the HO-1/Nrf2 axis can overcome resistance in biologically defined subgroups.

## Figures and Tables

**Table 1:** Demographics.

Age Years, mean (SD)	63.8 (11.13)
Male Genger, n, (%)	35 (60.3)
Urban living environment, n, (%)	36 (62.0)
ASA Score, n, (%) 0 1 2	12 (20.6) 16 (27.5) 30 (51.7)
Liver Cirhosis	45 (77.5)
CHILD-PUGH SCORE A B C	36 (62.0) 8 (13.7) 1 (1.72)
MILAN CRITERIA EXTRAMILAN	34 (58.6) 24 (41.3)
Aetiology, n, (%) HBV HCV Toxic	6 (10.3) 33 (56.9) 7 (12.1)

**Table 2:** Univariate analysis of HO-1 expression and clinicopathological parameters.

Clinicopathological Parameters		n	HO-1 Expression		*p*-Value	Cramer’s V
			Low	High		
**Gender**	male	49	36 (62.1%)	13 (22.4%%)	0.69	0.055
	female	9	6 (10.3%)	3 (5.2%)		
**Age** **(years)**	<50	5	3 (5.2%)	2 (3.4%)	0.60	0.085
	>50	53	39 (67.2%)	14 (24.1%)		
**HCV etiology**	absent	25	23 (39.7%)	2 (3.4%)	**0.004**	0.381
	present	33	19 (32.8%)	14 (24.1%)		
**HBV etiology**	absent	52	36 (62.1%)	16 (27.8%)	0.17	0.210
	present	6	6 (10.3%)	0 (0%)		
**Alcohol-related** **etiology**	absent	51	36 (62.1%)	15 (25.9%)	0.66	0.110
	present	7	6 (10.3%)	1 (1.7%)		
**Serum AFP** **(ng/mL)**	1–9	9	6 (10.3%)	3 (5.2%)	0.91	0.061
	10–99	35	26 (44.8%)	9 (15.5%)		
	>100	14	10 (17.2%)	4 (6.9%)		
**Tumor size** **(cm)**	0-3	50	36 (62.1%)	14 (24.1%)	0.30	0.237
	4/5	7	6 (10.3%)	1 (1.7%)		
	>5	1	0 (0%)	1 (1.7%)		
**Tumor number**	0–3	16	12 (20.7%)	4 (6.9%)	0.86	0.076
	4/5	15	10 (17.2%)	5 (8.6%)		
	>5	27	20 (34.5%)	7 (12.1%)		
**Milan criteria for liver transplantation**	eligible	31	26 (44.8%)	5 (8.6%)	**0.03**	0.275
	noneligible	27	16 (27.6%)	11 (19%)		
**BCLC grading**	Stage A	48	35 (60.3%)	13 (22.4%)	1.000	0.025
	Stage B	10	7 (12.1%)	3 (5.2%)		
**Perioperative anticoagulant therapy**	absent	31	27 (46.6%)	4 (6.9%)	**0.007**	0.352
	present	27	15 (25.9%)	12 (20.7%)		
**Antiviral therapy**	absent	52	38 (65.5%)	14 (24.1%)	0.66	0.044
	present	6	4 (6.9%)	2 (3.4%)		
**Perioperative TACE**	absent	54	38 (65.5%)	16 (27.6%)	0.56	0.168
	present	4	4 (6.9%)	0 (0%)		
**Postoperative TACE**	absent	55	40 (69%)	15 (25.9%)	1.000	0.030
	present	3	2 (3.4%)	1 (1.7%)		
**Chemotherapy**	absent	43	30 (51.7%)	13 (22.4%)	0.52	0.100
	present	15	12 (20.7%)	3 (5.2%)		
**Liver cirrhosis**	Absent	18	14 (24.1%)	4 (6.9%)	0.75	0.081
	Present	40	28 (48.3%)	12 (20.7%)		

**Table 3:** Univariate analysis of HO-1 expression and histopathological parameters.

Pathological Parameters		n	HO-1 Expression		*p*-Value	Cramer’s V
			Low	High		
**Trabecular growth pattern**	absent	4	2 (3.4%)	2 (3.4%)	0.30	0.136
	present	54	40 (69%)	14 (24.1%)		
**Solid growth pattern**	absent	31	23 (39.7%)	8 (13.8%)	0.77	0.043
	present	27	19 (32.8%)	8 (13.8%)		
**Pseudoglandular growth pattern**	absent	17	9 (15.5%)	8 (13.8%)	**0.05**	0.281
	present	41	33 (56.9%)	8 (13.8%)		
**Histological grade**	well differentiated	8	7 (12.1%)	1 (1.7%)	0.71	0.136
	moderately differentiated	43	30 (51.7%)	13 (22.4%)		
	poorly differentiated	7	5 (8.6%)	2 (3.4%)		
**Tumor stage**	1	19	17 (29.3%)	2 (3.4%)	0.08	0.339
	2	29	17 (29.3%)	12 (20.7%)		
	3	7	5 (8.6%)	2 (3.4%)		
	4	3	3 (5.2%)	0 (0%)		
**Lymphatic invasion**	absent	51	38 (65.5%)	13 (22.4%)	0.38	0.127
	present	7	4 (6.9%)	3 (5.2%)		
**Vascular invasion**	absent	29	25 (43.1%)	4 (6.9%)	**0.02**	0.309
	present	29	17 (29.3%)	12 (20.7%)		

**Table 4:** Youden index interpretation.

Youden Index J	0.2759
Associated criterion	>1
Sensitivity	41.38
Specificity	86.21

**Table 5:** Survival analysis based on HO-1 expression.

	Estimate	Std. Error	95% Confidence Interval	
			Lower Bound	Upper Bound
Negative	40.829	5.996	29.077	52.581
Positive	37.607	6.147	25.560	49.655
Overall	38.606	4.259	30.258	46.953

**Table 6:** Correlations of HO -1 expression and disease recurrence and OS.

		Ho-1	Overall Survival	Disease Recurrence
Overall survival	Pearson Correlation	−0.061	1	−0.047
	Sig. (2-tailed)	0.650		0.728
	N	58	58	58
Disease recurrence	Pearson Correlation	0.014	−0.047	1
	Sig. (2-tailed)	0.915	0.728	
	N	58	58	58

**Figure A1:**
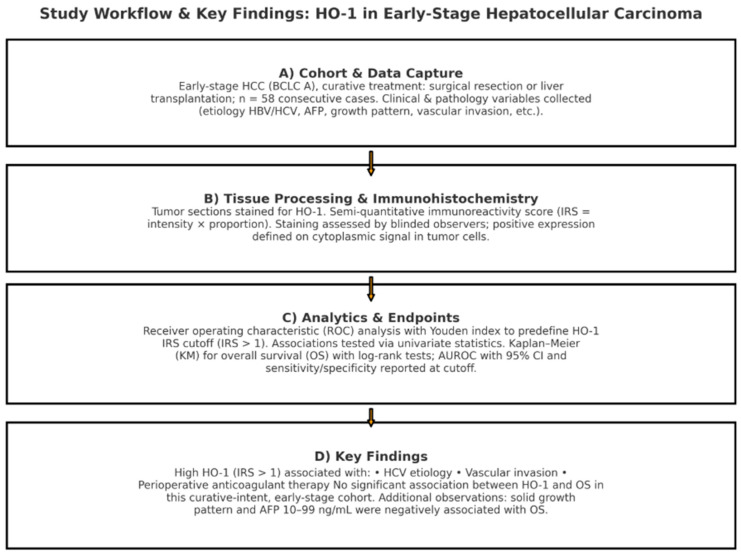
Study workflow and key findings. (**A**) Cohort & data capture: early-stage HCC (BCLC A) treated with resection or transplantation; n = 58; clinical and pathology variables collected (etiology HBV/HCV, AFP, growth pattern, vascular invasion). (**B**) Tissue/IHC: HO-1 immunohistochemistry; semi-quantitative immunoreactivity score (IRS = intensity × proportion), blinded scoring on cytoplasmic tumor staining. (**C**) Analytics: ROC analysis with Youden index predefined the HO-1 threshold (IRS > 1); AUROC with 95% CI and sensitivity/specificity reported; univariate associations; Kaplan–Meier for overall survival (OS) with log-rank p. (**D**) Key findings: high HO-1 associated with HCV etiology, vascular invasion, and perioperative anticoagulant therapy; no significant association between HO-1 and OS; solid growth pattern and AFP 10–99 ng/mL were associated with worse OS.

## Data Availability

Due to GDPR and institutional laws we cannot provide the datasets.

## Abbreviations

HCC	hepatocellular carcinoma
HO-1	heme oxygenase-1
HBV	hepatitis B virus
OS	overall survival
HCV	hepatitis C virus
NAFLD	nonalcoholic fatty liver disease
NASH	nonalcoholic steato-hepatitis
IAPs	inhibitors of apoptosis proteins
BCLC	Barcelona Clinic Liver Cancer
TACE	trans arterial chemoembolization
IRS	immunoreactivity score
ROC	receiver operating characteristic
AUC	area under the ROC curve
EMT	epithelial-to-mesenchymal transition.
VEGF	vascular endothelial growth factor
